# Upper Body Movement Symmetry in Reining Quarter Horses during Trot In-Hand, on the Lunge and during Ridden Exercise

**DOI:** 10.3390/ani12050596

**Published:** 2022-02-27

**Authors:** Thilo Pfau, W. Michael Scott, Tabitha Sternberg Allen

**Affiliations:** 1 Department of Clinical Science and Services, The Royal Veterinary College, Hawkshead Lane, North Mymms, Hatfield AL9 7TA, UK; tsternberg7@rvc.ac.uk; 2 Faculty of Veterinary Medicine, University of Calgary, 2500 University Dr NW, Calgary, AB T2N 1N4, Canada; wmichael.scott@ucalgary.ca; 3 Faculty of Kinesiology, University of Calgary, 2500 University Dr NW, Calgary, AB T2N 1N4, Canada

**Keywords:** movement symmetry, straight-line, lunge, ridden exercise, Quarter Horse

## Abstract

**Simple Summary:**

Veterinary lameness examinations often include assessment of the horse while ridden. Movement symmetry data are commonly used to aid veterinary decision making in lame horses. Reference data are available for horses ridden in ‘English style’, but not specifically for horses ridden in ‘Western style’ with considerable differences in head–neck position, tack and riding style. Upper body movement symmetry was measured in thirty reining Quarter Horses (QHs) during trot in-hand, lunged and ridden by one experienced rider on reining-purpose riding surfaces. Movement was marginally more symmetrical in the ridden horse, with small differences of 1–5 mm. Typical for soft surfaces, movement asymmetry indicated reduced weight support with the limb on the inside of the circle and reduced pushoff with the opposite limb. The small but significant asymmetry differences between in-hand and ridden exercise in QHs ridden in ‘Western style’ necessitate further studies in lame horses. Particular focus should be put on investigating the role of ridden exercise for highlighting differences between specific lameness causes and weight bearing and pushoff forces with the lame limb on the inside or outside of the circle.

**Abstract:**

Veterinary lameness examinations often comprise assessing ridden horses. Quantitative movement symmetry measurements can aid evidence-based decision making. While these are available for ‘English’ style riding, they are not for ‘Western’ style riding. This quantitative observational study quantified movement symmetry in reining Quarter Horses (QHs). Movement symmetry of the head, withers and sacrum (differences between minima, maxima and upward amplitudes) were quantified with inertial sensors in *N* = 30 medium/high level reining QHs during trot in-hand, on the lunge and ridden by one experienced rider (straight-line/circles) on reining-purpose riding surfaces. Mixed linear models for movement symmetry assessed the effects of ridden exercise and movement direction (fixed factors), stride time (covariate) and horse (random factor): single factors and two-way interactions with Bonferroni correction at *p* < 0.05. Three withers and pelvic parameters showed marginally more symmetrical movement when ridden (*p* ≤ 0.044; 1–5 mm differences). Three withers, three sacrum and one head parameter were significantly affected by movement direction (all *p* ≤ 0.026), five showed increased asymmetry on the inside rein, and two, quantifying vertical displacement maximum difference, showed the opposite. Riding QHs in ‘Western’ style showed small movement symmetry differences. Circular exercise confirmed increases in weight bearing asymmetry on the inside rein and in pushoff asymmetry on the outside rein. This should be further investigated for differentiating between different causes of lameness.

## 1. Introduction

In the horse, the veterinary lameness examination consists of a logical, structured process aiming at identifying any impairments affecting the locomotor apparatus and locating the source of the impairment [1]. A large part of the lameness examination encompasses the assessment of the horse in motion [2]. The importance of ridden exercise as part of the dynamic examination for lameness and poor performance is increasingly being recognized [3].

Modern gait analysis techniques, in particular inertial measurement units (IMUs) are ideally suited for quantitative measurements aiding veterinary decision making: they offer maximum freedom of movement through medium range wireless links or onboard data storage and do not require any sensors on the limbs of the horses [4,5,6,7,8,9]. They also do not require line of sight and can thus be used to quantify multidimensional ranges of motion of the thoraco-lumbo-sacral area and can be attached under the saddle in the ridden horse when required [10,11,12].

Owing to the flexibility afforded by IMU-based gait analysis systems, a number of studies have investigated their use in clinical studies and/or for quantifying the effect of clinically relevant exercises such as flexion tests [13,14], before/after diagnostic analgesia [15,16,17,18,19,20], during lunging [21,22,23,24,25,26] or during movement under the rider [11,27,28,29]. Quantitative information is needed for creating databases, facilitating an evidence-based approach for veterinary decision making for lameness and poor performance examinations.

To the authors’ knowledge, no studies have been performed quantifying movement symmetry in Quarter Horses (QHs) ridden in ‘Western style’: in addition to the difference in riding style and equipment, QHs are used for a variety of disciplines with the musculoskeletal system put under different loads and experiencing different injury distributions [30,31,32,33,34,35]. This warrants further studies into the QH and discipline-specific changes in movement symmetry experienced between straight-line trot, lunging and ridden exercise under ‘real-life’ conditions in comparison to what is known from different types of horses and riding styles. In agreement with previous studies in non-QHs quantifying movement asymmetry during circular movement [21,22,23,24,27], we hypothesized that movement asymmetries would be exacerbated on the lunge, in particular when the limb associated with the pre-existing movement asymmetry is on the inside of the circle where circle-induced [25] and pre-existing asymmetry effects are often summative [36] and that the addition of a rider would further exacerbate this [27,28].

## 2. Materials and Methods

Ethical approval was granted by the Clinical Research Ethical Review Board (CRERB) at the Royal Veterinary College, under unique reference number (URN) 2021-2027-3. Signed consent was provided by the trainers of the horses as authorized agents by the horse owners.

### 2.1. Horses

A convenience sample of 30 American QHs were included in the study, which were in training in their respective age category at an intermediate to high level in reining and were consistently ridden at least five times per week. All horses were judged, by their trainers, to be working comfortably. There were 13 geldings, 4 stallions and 13 mares, the mean age was 5.8 years (range: 3–14), and the mean height at the withers was 1.48 m (range: 1.40 to 1.58 m). Data were collected from three training facilities in the United States, with one located in Pilot Point, Texas, and two in Scottsdale, Arizona.

### 2.2. Gait Analysis Sensors

Each horse was equipped with a wireless inertial measurement unit (IMU) gait analysis system (Xsens DOT, tri-axial gyroscope, accelerometer and magnetometer, +/− 2000 deg s^−1^, +/− 16 gravity, +/− 8 Gauss), consisting of three IMUs attached with double sided tape and custom-made Velcro pouches to the horse. Anatomical landmarks were used to place the sensors over the poll (centred between the ears at the highest point), over the withers (in the region of the forth to sixth thoracic vertebrae) and on the sacrum (centred between the tubera sacrale). The sensors were synchronized with a dedicated iOS smartphone app (Xsens DOT Application, Xsens Technologies B.V., Enschede, The Netherlands) communicating with an iPhone8 (Apple Inc., Cupertino, CA, USA). Data collection was initiated manually, with an operator starting the recording from the app communicating with the IMUs via a Bluetooth Low Energy (BLE) connection logging data onto the onboard memory at a sample rate of 60 Hz per individual channel.

### 2.3. Assessment Conditions

Horses were initially allowed to warm up (walk, trot and canter; without tack) on the lunge for approximately five minutes. For assessment, each horse performed a pre-defined exercise routine comprising seven conditions: (1) walk in-hand, (2) trot in-hand in a straight line, (3) trot on the lunge on the left rein and (4) on the right rein, (5) trot ridden in a straight line, (6) on the left rein and (7) on the right rein. Circle diameters for both lunge and ridden exercises were approximately 15 m. The order of exercises was randomly allocated and varied between horses. The aim of the data collection was to collect approximately 25–30 strides [4] during steady state locomotion, in order to provide a representative sample of each horse’s gait for each of the seven assessment conditions. In the event that a horse showed excessive head movement or broke into a different gait, the condition was repeated until a data collection free of unwanted behaviors was obtained. All exercises were performed on soft, reining-purposed arena surface (sand on a clay base). In order to minimize the effect of different riders [37], the same rider (height 1.58 m, body mass 63 kg, holding the reins in the left hand) and tack (16 kg) was used for each horse. The tack included splint boots on the forelimbs, a western reining style saddle and saddle pad, and a bridle with either a small port shanked bit or loose ring snaffle bit (depending on the age of the horse).

### 2.4. Data Processing

Data from each sensor including orientation (Euler angles in degrees), tri-axial acceleration in ms^−2^, tri-axial angular velocity in degrees s^−1^ and tri-axial magnetic field strength in Gauss were downloaded from the onboard memory in between data collection of subsequent horses, onto a laptop computer (Microsoft Surface Go2) running Microsoft Windows 10Pro. Custom written software (MATLAB, The MathWorks Inc., Natick, MA, USA) was used to extract movement symmetry values from vertical displacement of trot data, following published procedures for the integration process [38] and for stride segmentation from continuous data into individual strides [39]. Walk data were not analyzed in this study. 

Trot movement symmetry measures for each IMU included differences in displacement minima, displacement maxima and upward movement amplitudes between stride halves, parameters which have previously been associated with asymmetry in force production [40,41]. In particular, differences between displacement minima are associated with differences in vertical peak force (‘weight bearing’) between contralateral limbs [40,41], and differences between displacement maxima (in horses without notable minimum differences) have been related to force asymmetries in the second half of stance (‘pushoff’).

Movement asymmetry parameters were quantified for each stride cycle, and then median values were calculated across all available strides per exercise condition. The resulting nine asymmetry parameters—three for head displacement (HDmin, HDmax, HDup), three for withers displacement (WDmin, WDmax, WDup) and three for pelvic displacement (PDmin, PDmax, PDup) [22]—were tabulated together with information about stride time (retained from the stride segmentation process [39]), exercise condition (in-hand vs. ridden) and movement direction (straight-line/left/right). The following sign convention was applied to label the movement asymmetry values: positive values were assigned to asymmetries typical in direction for horses with right forelimb (RF) or right hind limb (RH) lameness, i.e., asymmetry traces showing reduced movement during/after RF (or RH) stance. Negative values were assigned to asymmetries typical in direction for horses with left forelimb (LF) or left hind limb (LH) lameness, i.e., showing movement traces with reduced movement during/after LF (or LH) stance.

In order to illustrate the magnitude and direction of pre-existing movement asymmetries in the study cohort of *N* = 30 reining QHs, box plots were created for in-hand straight-line trot and for lunge exercise on the left and right rein.

### 2.5. Data Normalization

The following data normalization was implemented to more effectively utilize data from a mixture of horses showing varying amounts of pre-existing left and right-sided movement asymmetries. Pre-existing ‘baseline’ asymmetries were defined as the values measured during in-hand trot, on the straight, with each asymmetry parameter considered separately. For horses with a negative baseline asymmetry value, all values for that particular parameter were inverted (i.e., for all six trot exercise conditions) by multiplying the originally recorded value by negative one. In the first instance, this resulted in all normalized baseline, straight-line values being positive. Secondly, asymmetries for the remaining exercise conditions were then expressed in relation to the positive baseline value, i.e., the sign expressing whether the asymmetry was of the same direction (positive sign) or of the opposite direction (negative sign) compared to the baseline asymmetry independent of whether the baseline condition indicated a left or right-sided asymmetry.

In addition, the directional labels of lunge or circular ridden exercise conditions were expressed in relation to the baseline asymmetry: for horses with left-sided baseline asymmetry, ‘left’ rein labels were relabeled as ‘inside’, and ‘right’ rein labels as ‘outside’ (and vice versa for horses with a right-sided baseline asymmetry). Thus, the exercise condition of a horse showing a movement pattern indicative of reduced weight bearing (or pushoff) produced with a left limb and trotting on a left circle was labeled as being on the ‘inside rein’, i.e., with the limb associated with reduced weight bearing (or pushoff) force production on the inside of the circle (see Table S1). This is analogous to the process during a lameness examination, whereby the severity of the lameness is judged by the veterinarian on both the ‘inside’ and ‘outside’ of a circle, i.e., when the horse circles with the lame limb on the inside or the outside of the circle.

### 2.6. Statistical Testing

Data were analyzed using SPSS statistical software (Version 28). Quantitative movement symmetry values of head, withers and pelvis were compared between different in-hand, lunge and riding conditions with mixed model analysis. Separate models were created for each normalized movement asymmetry variable as the dependent variable with fixed factors as exercise condition (in-hand vs. ridden; ‘IHvsRID’) and rein (straight; inside; outside) and with stride time as a covariate. Horse ID was included as a random factor; the randomized order of the exercises was not modelled. The level of significance was set to *p* < 0.05 throughout. Bonferroni corrections were applied for pairwise comparisons for factors with more than two categories (single factor: rein) and for 2-way interactions: IHvsRID × rein; IHvsRID × stride time; rein × stride time. For ease of illustrating results of the post hoc analysis of two-way interactions involving the fixed covariate stride time, a categorical stride time variable was introduced (with values ‘low’ and ‘high’) with the median value (736 ms) used as a cut off between the two categories. Conditions associated with a ‘low’ value, i.e., showing a reduced stride duration, are indicative of a faster trot, conditions associated with a ‘high’ value, i.e., a higher stride duration, are indicative of a slower trot [42].

Initial full mixed model analysis with single factors and all two-way interactions were run and then pruned such that two-way interactions with *p* > 0.1 were eliminated from the final models. Estimated marginal means were investigated to demonstrate the size of any differences and for judging the biological significance in terms of which condition (or conditions) showed increased or decreased movement asymmetry.

Model fit of the final models was evaluated by plotting histograms of model residuals and visual evaluation against a normal distribution.

## 3. Results

### 3.1. Number of Strides and Stride Time

An average of 25 strides (range 4 to 53) were analyzed for each of the six in-hand and ridden trot exercise conditions with a mean stride time of 737 ms (range 609 ms to 877 ms). For non-ridden exercise, stride time averaged at 697 ms (range 609 ms to 778 ms), for ridden exercise a mean stride time of 777 ms was measured (range 683 ms to 877 ms). The average number of strides analyzed was 19 for straight-line exercise, 29 strides on the left circle and 27 strides on the right circle.

### 3.2. Baseline and Lunge Movement Symmetry Prior to Normalization

**Figure 1:**
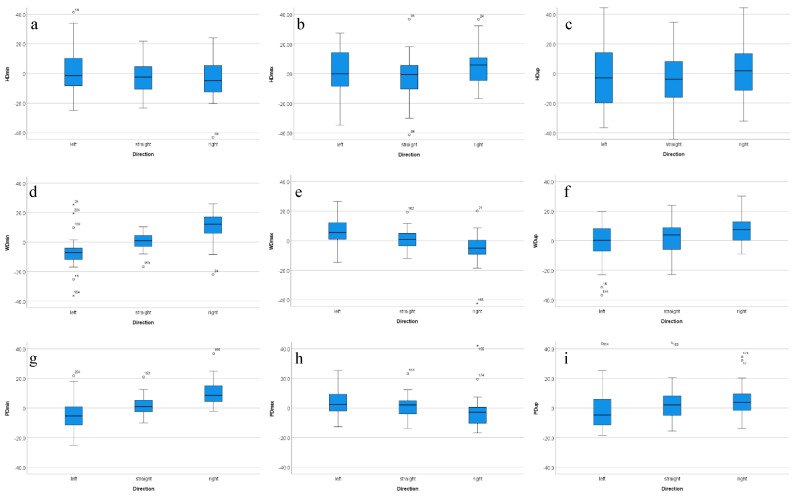
Head, withers and sacrum movement symmetry values (in mm) for *N* = 30 reining QHs for baseline (‘straight’: in-hand, straight-line) and left and right lunge (‘left’; ‘right’) exercise collected with a 3-sensor inertial sensor gait analysis system on reining-purpose arena footing. Positive asymmetry values indicate ‘right asymmetrical’ movement, negative values indicate ‘left asymmetrical’ movement. (**a**) HDmin, (**b**) HDmax, (**c**) HDup, (**d**) WDmin, (**e**) WDmax, (**f**) WDup, (**g**) PDmin, (**h**) PDmax, (**i**) PDup. Outliers ‘°’ defined as data points more extreme than first quartile −1.5 times interquartile range or more extreme than third quartile +1.5 times interquartile range. Extreme outliers ‘*’ defined as data points more extreme than first quartile −3 times interquartile range or more extreme than third quartile +3 times interquartile range.

Descriptive statistics for baseline symmetry values for vertical displacement of head, withers and sacrum calculated for *N* = 30 reining QHs from straight-line, in-hand trot as well as for left and right lunge exercise are presented in Table 1 and illustrated in Figure 1a–f.

In general, median asymmetry values across all horses for straight-line trot are close to zero ranging from −3.90 mm to +3.90 mm. Differences between left and right rein median values showed values of 8 mm or less, with the exception of WDmin (19.1 mm difference), WDmax (10.6 mm) and PDmin (13.9 mm difference). See column ‘50th’ in Table 1 for median values for left rein, right rein and straight-line data.

On the straight, all asymmetry parameters ranged (as quantified through investigating interquartile ranges, see columns 25th and 75th in Table 1) from negative (left-sided) to positive (right-sided) values across horses. Variation on the straight was generally high for head movement (interquartile ranges between 15.5 mm and 25.8 mm) and had lower values for withers and sacrum (interquartile ranges from 7.6 mm to 15.1 mm).

Box plots and interquartile ranges for withers and sacrum asymmetry parameters derived from differences between minima and between upward amplitudes illustrate a tendency for increased right asymmetrical movement, i.e., positive values, on the right rein and increased left asymmetrical movement, i.e., negative values, on the left rein (WDmin, WDup, PDmin and PDup; Figure 1, Table 1). WDmax and PDmax show the opposite tendency indicating increased right asymmetrical movement on the left rein and vice versa. Head movement asymmetry appears least consistently affected by movement direction.

### 3.3. Effects of Movement Direction, Ridden Exercise and Stride Time

Mixed model analysis showed that four normalized parameters were significantly affected by ridden exercise, seven by movement direction and two by stride time (see Table 2).

None of the head parameters were affected by ridden exercise (all *p* ≥ 0.296) while both withers and sacrum parameters related to minimum and upward movement amplitude differences (WDmin, WDup, PDmin, PDup) were significantly affected by ridden exercise (all *p* ≤ 0.044). 

All three withers and sacrum parameters were affected by movement direction (all *p* ≤ 0.004), while HDup (*p* = 0.026) was the only head parameter affected by movement direction. 

Two head movement parameters (HDmax: *p* = 0.042 and HDup: *p* = 0.007) were affected by stride time, none of the withers and sacrum parameters were affected by stride time (all *p* ≥ 0.126).

Of the four parameters affected by ridden exercise, three parameters (WDmin: 4.48 mm vs. 1.25 mm; WDup: 8.54 mm vs. 3.38 mm; PDup: 3.98 mm vs. 2.55 mm) showed reduced asymmetry during ridden exercise compared to trot in-hand. PDmin showed the opposite effect with 1.60 mm asymmetry during in-hand exercise and 1.93 mm during ridden trot (see Table 3).

**Figure 2:**
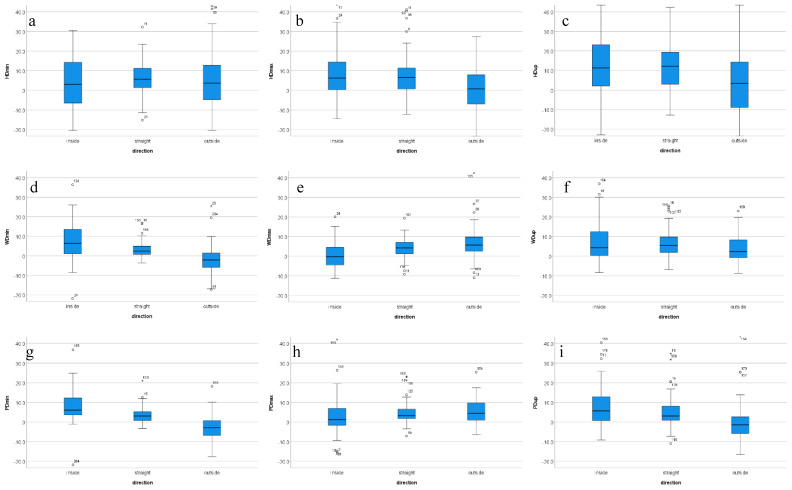
Normalized movement asymmetry values (in mm) for *N* = 30 reining QHs as a function of movement direction for trot on reining type arena surfaces during in-hand and ridden exercise for straight-line and circular movement. Positive asymmetry values indicate movement asymmetry in the direction of the measured baseline asymmetry, negative values indicate movement asymmetry opposite in direction to the measured baseline asymmetry. (**a**) HDmin, (**b**) HDmax, (**c**) HDup, (**d**) WDmin, (**e**) WDmax, (**f**) WDup, (**g**) PDmin, (**h**) PDmax, (**i**) PDup. Outliers ‘°’ defined as data points more extreme than first quartile −1.5 times interquartile range or more extreme than third quartile +1.5 times interquartile range. Extreme outliers ‘*’ defined as data points more extreme than first quartile −3 times interquartile range or more extreme than third quartile +3 times interquartile range. Outliers ‘°’ defined as data points more extreme than first quartile −1.5 times interquartile range or more extreme than third quartile +1.5 times interquartile range. Extreme outliers ‘*’ defined as data points more extreme than first quartile −3 times interquartile range or more extreme than third quartile +3 times interquartile range.

For five of the seven parameters affected by movement direction (HDup, WDmin, WDup, PDmin, PDup), the highest movement asymmetry value was measured on the inside rein and the lowest on the outside rein (Table 4, Figure 2). Three of these (WDmin, PDmin and PDup) registered a negative asymmetry value—i.e., an asymmetry in the opposite direction of the baseline asymmetry—on the outside rein. For the remaining two of the seven parameters (WDmax and PDmax), the highest asymmetry value was observed on the outside rein, the lowest value on the inside rein.

Estimated marginal mean values and Bonferroni-corrected *p*-values for the significantly different pairs are presented in Table 4. Head movement in general appears less affected by movement direction (exception: HDup with decreased asymmetry on the outside rein). WDmin, PDmin and PDup are changing asymmetry direction on the outside rein (i.e., take negative values). WDmax and PDmax show increasingly positive values from the inside rein, via straight-line to the outside rein.

### 3.4. Two-Way Interactions between In-Hand Versus Ridden Exercise, Movement Direction and Stride Time

After model reduction, seven 2-way interactions remained in the final models, five of which returned significant *p*-values, two for sacral movement, three for withers movement, none for head movement.

#### 3.4.1. Sacral Movement Asymmetry

**Figure 3:**
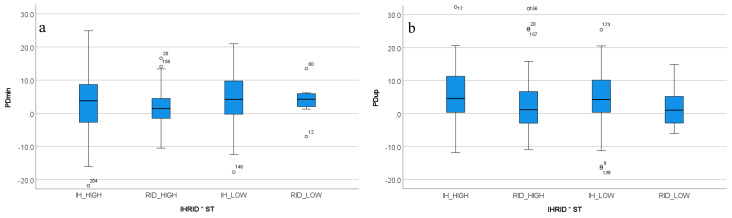
Box plots for significant two-way interactions for (**a**) PDmin and (**b**) PDup. PDmin and PDup are the only two variables to be significantly affected by the two-way interaction of in-hand versus ridden exercise with stride time. For both sacral asymmetry parameters there is a tendency for the ridden exercise to show lower asymmetry values (closer to zero). All movement asymmetry parameters are shown in mm. PDmin: minimum difference for vertical sacral movement; PDup: upward movement amplitude difference for vertical sacral movement; IH: in-hand exercise rein; RID: ridden exercise; HIGH: higher than average stride time; LOW: lower than average stride time.

Two sacral movement asymmetry parameters (PDmin: *p* = 0.042; PDup: *p* = 0.012) showed significant interactions between in-hand versus ridden exercise and stride time (Figure 3). For both PDmin and PDup, the highest asymmetry value (EMM: PDmin 3.87 mm, PDup 7.42 mm) was recorded for in-hand exercise combined with lower than average stride time (i.e., faster trot). The lowest asymmetry value (EMM: PDmin 0.77 mm; PDup −0.39 mm) was found for ridden exercise for lower than average stride time (i.e., faster trot).

For PDmin, Bonferroni-corrected pairwise comparisons did not reveal any significant pairwise differences (all *p* ≥ 0.43). For PDup, two Bonferroni-corrected pairwise significant differences were found, both involving in-hand exercise combined with lower than average stride time (IH_LOW), one in comparison to ridden exercise at higher than average stride time (RID_HIGH, *p* = 0.002) and the other in comparison to ridden exercise at lower than average stride time (RID_LOW, *p* = 0.038).

#### 3.4.2. Withers Movement Asymmetry

**Figure 4:**
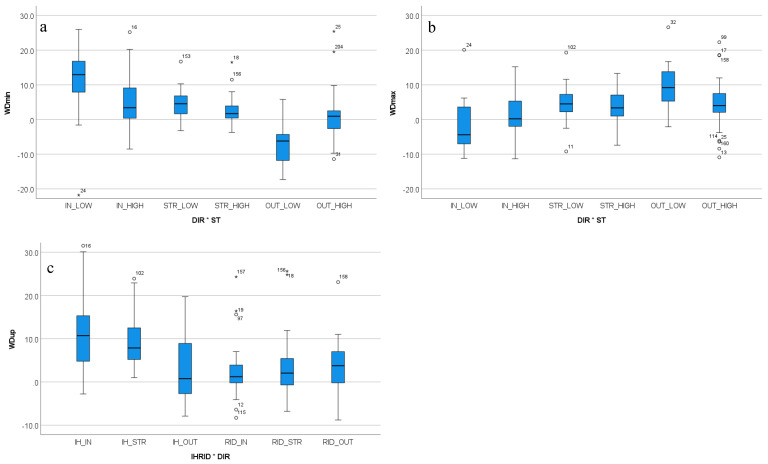
Box plots for significant two-way interactions for withers movement asymmetry (**a**) WDmin, (**b**) WDmax, (**c**) WDup. WDmin and WDmax are significantly affected by the two-way interaction of movement direction with stride time, WDup is significantly affected by the two-way interaction of in-hand versus ridden exercise with movement direction. All movement asymmetry parameters are shown in mm. WDmin: minimum difference for vertical withers movement; WDmax: maximum difference for vertical withers movement; WDup: upward movement amplitude difference for vertical withers movement; IN: inside rein; OUT: outside rein; HIGH: higher than average stride time; LOW: lower than average stride time. Outliers ‘°’ defined as data points more extreme than first quartile −1.5 times interquartile range or more extreme than third quartile +1.5 times interquartile range. Extreme outliers ‘*’ defined as data points more extreme than first quartile −3 times interquartile range or more extreme than third quartile +3 times interquartile range.

Three withers movement asymmetry parameters showed significant two-way interactions (Figure 4). WDmin and WDmax were significantly affected by the combination of movement direction and stride time (both *p* < 0.001). WDup was significantly affected by the combination of in-hand versus ridden exercise and movement direction (*p* < 0.001).

For WDmin, the highest amount of movement asymmetry (EMM: 9.89 mm) was found for trot on the inside rein with lower than average stride time (IN_LOW), while the lowest (only negative value (EMM: −9.70 mm) was found for trot on the outside rein also with lower than average stride time (OUT_LOW). These two conditions are also involved in the highest number of pairwise significant differences: five for outside rein and lower than average stride time (OUT_LOW) and four for inside rein and lower than average stride time (IN_LOW).

For WDmax, the most asymmetrical movement (EMM: 10.10 mm) is shown on the outside rein combined with lower than average stride time (OUT_LOW) and the lowest (only negative) value (EMM: −2.43 mm) was found for trot on the inside rein combined with lower than average stride time (IN_LOW). Identical to the pairwise differences reported for WDmin, these two conditions are also involved in the highest number of pairwise significant differences: five for outside rein, low stride time (OUT_LOW) and four for inside rein, low stride time (IN_LOW).

For WDup, the most asymmetrical movement (EMM: 12.10 mm) was found for trot on the inside in-hand (IH_IN), and the most symmetrical movement (lowest positive value, EMM: 2.52 mm) was found for ridden exercise also on the inside rein (RID_IN). Exercise in-hand on the straight (IH_STR) as well as exercise in-hand on the inside rein (IH_IN) showed Bonferroni-corrected pairwise significant differences to all other exercises but not between each other.

## 4. Discussion

To the authors’ knowledge, this is the first study reporting movement asymmetry values in reining QHs during both in-hand exercise and ridden in ‘Western style’ with a previous study including QHs reporting movement asymmetries for a straight-line and on the lunge (but not ridden) across different breeds [24]

### 4.1. Ridden Exercise

Previous studies investigating movement asymmetry in the context of ridden exercise have concentrated on horses ridden in ‘English style’ [27,28,43,44,45]. In our study, there was a tendency for ridden exercise to result in more symmetrical movement across withers and pelvis with three parameters (WDmin, WDup and PDup) showing this effect, while one parameter (PDmin) indicated the opposite effect. However, a more differentiated view is needed here: the differences, particularly for PDmin—i.e., indicating small changes in weight bearing between contralateral hind limbs [40]—are <1 mm, questioning their biological relevance, for example, in relation to the hind limb ‘lameness thresholds’ of 3 mm [46] typically applied to judging the outcome of diagnostic analgesia [15,18,19,20,47]. In addition, further factors that need to be taken into account are differences between sensor systems [48] as well as repeatability studies reporting intra- and inter-day variations [49,50].

Three of the investigated parameters feature significant two-way interactions. The two pelvic parameters show a significant two-way interaction with stride time. For PDmin, a parameter related to weight bearing differences [40], pairwise Bonferroni-corrected comparisons did not reveal any significant differences, questioning the <1 mm difference when investigating in-hand versus ridden exercise as a single factor for the particular rider studied here. For PDup on the other hand, a parameter related to pushoff asymmetries in the second half of stance [40], a reduction in asymmetry for the ridden exercise is confirmed by the pairwise comparisons, showing that in-hand exercise at faster trot (lower than average stride time) is significantly different from ridden trot at both speeds (lower and higher than average stride time). In the present study, reduced pelvic movement asymmetry during ridden exercise—best characterized as a ‘sitting jog’ typical for QHs—contrasts previous studies where, for example, Dressage riders in rising trot caused an increase in pelvic asymmetry on the straight [43,44,45] and on the circle [27,28]. Of course, rising trot imposes fundamentally different conditions on the horse during the rising and during the sitting part, while more similar conditions during the two halves of the stride can be expected in the ‘sitting jog’ used here, which is likely more similar to sitting trot and results in movement asymmetries similar to unridden exercise [27].

Licka and co-workers [43] indicated that there were differences between individual riders in sitting trot, with one significantly increasing pelvic asymmetry but not the other. In our study, with reining QHs ridden by one rider and with typical reining saddle and tack, overall, a reduction in pelvic movement asymmetry was observed. Whether or not this would hold true for other riders needs to be investigated further in relation to potential confounding factors relating to the rider or tack [10,11,27,37,43,51,52,53,54,55]. In this context, the reader is strongly advised to consider that our study only included one rider.

### 4.2. Movement Direction

In accordance with previous studies investigating circular movement [21,22,23,24,25,26], seven (out of nine) movement parameters were significantly affected by movement direction, generally reporting increased asymmetry on one rein and decreased asymmetry on the other rein, with asymmetry switching sides in some cases. In analogy to a previous study [22], movement asymmetry parameters expressing differences between vertical displacement minima or differences between upward amplitudes showed increased movement asymmetry on the inside rein (HDup, WDmin, WDup, PDmin, PDup). Movement asymmetry parameters expressing differences between vertical displacement maxima on the other hand (WDmax, PDmax) showed the opposite effect with increasing asymmetry on the outside rein. Making use of the association between movement and force asymmetry [40,41] one might argue that the peak force gets further reduced during stance of the inside limb and that the vertical impulse in the second half of the outside limb stance is reduced further. This may be useful for differentiating between horses aiming to reduce the peak force compared to horses aiming to reduce the muscle–tendon-aided pushoff [56,57] in the second half of stance.. However, first, the relationship between forces and movement asymmetry needs to be validated for movement on the circle and until then, assumptions about forces based on movement symmetry on the circle need to be viewed with caution.

Analyzing interactions between movement direction and stride time (i.e., speed, [42]) illustrate the potential of higher trotting speed (lower than average stride times) exacerbating pre-existing asymmetries [26], in particular for withers movement. Interestingly, WDmin and WDmax show almost opposite patterns: exercise on the inside rein at lower than average stride time creating the highest amount of WDmin and the lowest amount of WDmax. Exercise on the outside rein and higher speed showed the opposite effect. During straight-line trot, withers movement has been shown to successfully differentiate between forelimb and hind limb lame horses [58]. Our results (illustrated in Figure 2) show that head and withers movement asymmetry parameters show different patterns across the three movement directions, suggesting that the relationship between head and withers movement asymmetry changes and this should be further investigated in clinically lame horses.

### 4.3. Riding Style

The influence of a rider on a horse’s movement symmetry has been shown to be multifactorial, including elements such as the riding style, saddle fit, the rider’s weight, and head and neck position of the horse [11,27,28,37,44,45,52,59,60]. Although these factors could not be addressed individually in this study, when interpreting the results of the current study, contrasts between ‘English’ and ‘Western’ disciplines and their associations with these factors can be considered.

Reining horses differ in their training program and riding style compared to classically trained ‘English’ horses. Training objectives for reining include developing the sensitivity of the horse to the point that it can be ridden with light contact on the bit and loose reins held in one hand, as opposed to ‘English’ disciplines, where horses work with stronger, two-hand contact on the bit. Head carriage is therefore also different, with the ideal frame being consistently long and low (more similar to position 1 and 6 in [61]), compared to the higher, more flexed neck position in ‘English’ horses: force distribution shifted towards the thoracic limbs in long and low, or ‘free’ head and neck positions [61]. This shift might be useful for exacerbating forelimb force imbalances without influencing head movement and contribute to decision making in subtly lame horses.

In our study, the western ‘jog’ in the ridden horse appeared to be slower (increased stride times) than the trot that the horses performed freely on the lunge. Higher speeds have been reported to cause an increase in objectively measured asymmetry during circular movement [25,62] so this may reflect the inconsistencies when comparing results from studies based on possibly more impulsive Dressage horses that may have trotted at higher speeds as opposed to the slower and more relaxed reining horses. It would be interesting to directly quantify speed and not just stride time to further differentiate between speed-related movement changes and differences associated with specific head–neck positions.

Saddle fit and the weight supported by the equine back have been shown to influence gait [63] and should be considered. The tack utilized for ‘Western’ disciplines is different than that used for English disciplines, characterized by saddles that typically have a larger contact surface area and thicker pads. Western saddles differ in dimension and type depending on the intended discipline, which likely corresponds to significant differences in the conformation and size of different sub-populations within the Quarter Horse breed [64]. ‘English’ saddles are typically fitted to each individual horse and saddle fit and use of pads have been shown to influence saddle pressure distributions and motion variability [54,60,65,66]. Similar studies are required in the QH using ‘Western’ style tack.

### 4.4. Study Limitations

During data collection, the aim was to collect a minimum of 30 strides per exercise condition and horse with the intention to adequately capture average asymmetry values as well as stride to stride variability [4]. This was not always achieved. The person operating the phone starting and stopping data collection via the low energy Bluetooth link to the sensors (Xsens DOT) observed the exercise and was instructed to count strides. However, the automated stride extraction method [39] was combined with an additional empirical threshold based on variations in stride time (using a threshold of +/−15% from the median stride time). This resulted in elimination of strides at the beginning and end of each bout where the horses were accelerating or decelerating, as a result reducing the number of strides for some horses and conditions to less than 25. The automated elimination resulted in an average of 24.9 strides for straight-line data and of 18.9 strides on a circle, with some instances where less than 10 stride cycles were analysed (5 out of 180). The reason for this needs to be further investigated and alternative methods of restricting the analysis to steady state locomotion should be investigated.

For consistency, all exercise conditions were performed on the same, soft, reining-purposed arena surface across the three locations. Using a soft surface, particularly for the in-hand trot up on the straight is not representative of the process in a lameness work up, where horses are typically examined on both soft and firm surfaces [2], and, in particular, upper body movement asymmetry parameters extracted from circular movement have been shown to vary between different surfaces [21]. Our results should, hence, not be generalized to other surfaces.

## 5. Conclusions

This study is the first to report upper body movement symmetry in reining QHs during in-hand exercise, on the lunge and when ridden in ‘Western style’.

In our study, there was a tendency for ridden exercise to result in more symmetrical movement across the withers and pelvis. However, small differences question the biological relevance in the context of hind limb lameness, where a ‘lameness threshold’ of 3 mm [46] is typically applied.

The majority of movement parameters were significantly affected by movement direction, generally showing increased asymmetry for parameters comparing vertical minima or upward amplitudes when the horse is moving on the ‘inside rein’. Movement asymmetry parameters expressing differences between vertical displacement maxima showed increased asymmetry on the outside rein. Further studies with clinically lame horses and diagnostic analgesia should investigate whether there are differences between horses with different locomotor deficits.

Reining horses differ in their training program and riding style compared to classically trained ‘English’ horses. Further studies may want to investigate the influence of parameters such as ‘head carriage’, the western ‘jog’ under the rider and the specific tack used.

## Figures and Tables

**Table 1:** Percentiles for 6 movement symmetry measures (in mm) calculated from vertical displacement of head, withers and sacrum for in-hand, straight-line trot and left and right lunge exercise for *N* = 30 reining Quarter Horses. Highlighted are straight-line percentiles (bold) and median (50th percentile, bold) values.

		Percentiles						
Direction		5	10	25	50	75	90	95
**HDmin**	left	−21.1	−16.4	−9.1	−1.4	10.8	19.3	37.3
	straight	−21.4	−15.3	**−10.7**	**−2.4**	**4.9**	18.0	20.5
	right	−30.6	−19.9	−12.6	−4.9	6.1	16.8	20.5
**HDmax**	left	−31.0	−23.0	−9.5	−0.1	14.3	22.4	24.8
	straight	−34.9	−18.7	**−10.5**	**−0.5**	**5.6**	15.5	26.5
	right	−16.8	−14.3	−5.4	5.9	11.0	20.9	34.2
**HDup**	left	−36.4	−28.3	−20.0	−3.0	16.1	39.3	50.5
	straight	−45.6	−28.2	**−16.9**	**−3.9**	**9.0**	25.3	33.5
	right	−44.6	−25.0	−12.0	1.8	13.6	27.0	38.8
**WDmin**	left	−30.2	−16.9	−12.1	−7.1	−3.9	9.0	22.2
	straight	−11.9	−7.3	**−3.1**	**1.0**	**4.5**	6.8	8.9
	right	−14.5	−5.2	6.0	12.1	17.2	20.1	22.9
**WDmax**	left	−10.1	−6.4	1.0	5.6	12.5	16.5	24.2
	straight	−9.9	−7.7	**−3.7**	**0.9**	**5.2**	10.1	15.1
	right	−29.3	−13.1	−9.3	−5.0	1.2	8.0	13.8
**WDup**	left	−33.9	−22.2	−7.2	0.3	8.3	16.9	19.3
	straight	−20.9	−14.6	**−6.2**	**3.9**	**8.9**	12.9	18.0
	right	−26.6	−8.1	0.5	7.4	13.0	21.4	29.4
**PDmin**	left	−22.0	−14.1	−11.6	−5.4	1.2	9.3	19.8
	straight	−9.3	−7.8	**−2.7**	**1.0**	**5.5**	11.0	16.4
	right	−0.2	1.5	4.2	8.6	15.3	24.2	30.3
**PDmax**	left	−11.9	−11.0	−2.8	2.4	9.4	16.7	20.9
	straight	−13.2	−6.8	**−4.0**	**2.2**	**5.0**	8.7	17.3
	right	−16.7	−16.0	−10.7	−2.8	0.7	6.9	29.6
**PDup**	left	−17.4	−16.1	−11.5	−4.7	6.6	11.2	33.5
	straight	−15.1	−9.8	**−5.0**	**2.1**	**8.7**	20.1	31.2
	right	−11.8	−7.9	−1.7	3.8	9.9	31.1	54.4

**Table 2:** Overview of the results of the mixed model investigating the effect of in-hand versus ridden exercise (IHvsRID), of stride time (ST) and of movement direction (DIR). Presented are *p*-values for single factors IHvsRID, DIR and ST and their interactions (where relevant for each of the reduced models). Significant *p*-values (*p* < 0.05) in bold.

	IHvsRID	ST	DIR	IHvsRID*ST	IHvsRID*DIR	DIR*ST
**HDmin**	0.438	0.590	0.166	/	/	/
**HDmax**	0.999	**0.042**	0.052	/	0.055	0.085
**HDup**	0.296	**0.007**	**0.026**	/	/	/
**WDmin**	**0.022**	0.430	**<0.001**	/	/	**<0.001**
**WDmax**	0.264	0.463	**<0.001**	/	/	**<0.001**
**WDup**	**<0.001**	0.785	**0.004**	/	**<0.001**	/
**PDmin**	**0.044**	0.126	**<0.001**	**0.042**	/	/
**PDmax**	0.470	0.388	**<0.001**	/	/	/
**PDup**	**0.009**	0.146	**<0.001**	**0.012**	/	/

**Table 3:** Estimated marginal mean (EMM) values in mm for fixed factor IHvsRID (in-hand versus ridden exercise) for the four affected asymmetry parameters (see Table 2). All values in mm. The higher value for each asymmetry parameter is given in green, the lower value in red. Column ‘</>’ indicates whether the asymmetry for in-hand exercise is larger (‘>’) than for ridden exercise or smaller (‘<’).

IHvsRID	In-Hand	< / >	Ridden
	(mm)		(mm)
**WDmin**	4.48	**>**	1.25
**WDup**	8.54	**>**	3.38
**PDmin**	1.60	**<**	1.93
**PDup**	3.98	**>**	2.55

**Table 4:** Estimated marginal mean (EMM) values for fixed factor movement direction (DIR) for the seven affected asymmetry parameters. All values in mm. Values marked with same index have pairwise significant difference (Bonferroni). The highest value for each asymmetry parameter is given in green, the lowest value in red.

DIR	Inside Rein	Straight-Line	Outside Rein	Pairwise Comparison
				(Bonferroni)
**HDup**	12.2 ^1^	11.9	5.6 ^1^	^1^ *p* = 0.049
**WDmin**	7.5	3.4	−2.3	^1,2,3^ *p* < 0.001
**WDmax**	0.2 ^1,2^	4.1 ^1,3^	6.8 ^2,3^	^1,2,3^ *p* < 0.001
**WDup**	7.4 ^1^	6.3	4.2 ^1^	^1^ *p* = 0.003
**PDmin**	6.8 ^1,2^	2.7 ^1,3^	−4.2 ^2,3^	^1,2,3^ *p* < 0.001
**PDmax**	2.5 ^1,2^	4.8 ^1^	5.8 ^2^	^1^ *p* = 0.03; ^2^ *p* < 0.001
**PDup**	7.0 ^1,2^	4.2 ^1,3^	−1.3 ^2,3^	^1^ *p* = 0.059; ^2,3^ *p* < 0.001

## Data Availability

Data sets (unnormalized baseline asymmetry data and normalized movement symmetry data from in-hand, lunge and ridden exercise) analyzed in this study are accessible via FigShare at the following link https://figshare.com/s/052e01979d0353ec39e1 (accessed on 25 February 2022).

## Supplementary Materials

The following supporting information can be downloaded at: https://www.mdpi.com/article/10.3390/ani12050596/s1, Table S1: glossary of acronyms and terms.
